# Supplementary material for: Synchronous motion of the Easter mantle plume and the East Pacific Rise
Source: Nat Commun. 2024 Nov 17;15:9953. doi: 10.1038/s41467-024-54115-2 (PMC11570642; doi:10.1038/s41467-024-54115-2)
Supplement: Supplementary file 1 — Supplementary Information [file 41467_2024_54115_MOESM1_ESM.pdf]

Supplementary Information

Figure S1: Relative motion between the EPR and the Easter hotspot

Figure S2. DETOX-P2 map view centred on the Pacific LLVP

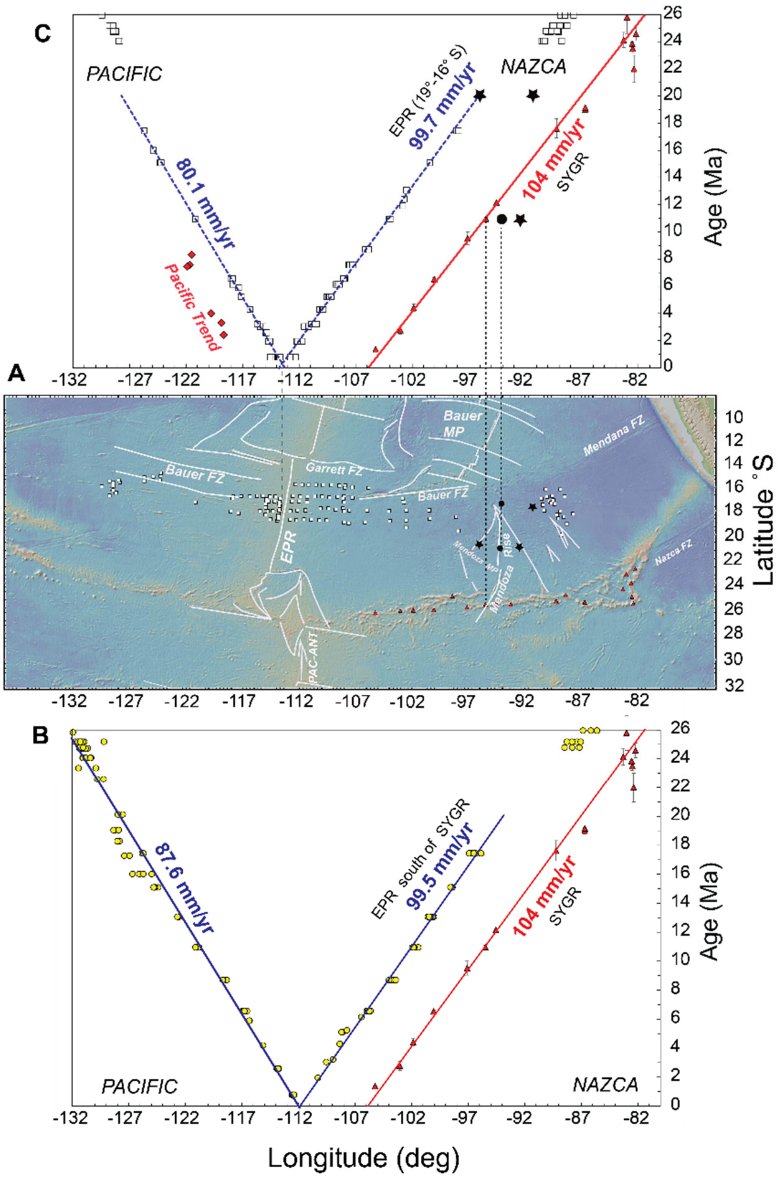

Figure S1. Correlation between the location of the EPR and the Easter hotspot.
A) Map showing the location of magnetic anomaly picks since anomaly C8n.1n (25.951 Ma) on both the Nazca and Pacific sides of the EPR. Star symbols represent picks linked to the propagation of the Mendoza Rise, while black dots indicate the youngest anomalies associated with the Mendoza Rise. Interpretation of the structure of the Mendoza Microplate and other features is adapted from Liu (1996). Additional details are as in Figure 6. **B**) Magnetic anomaly ages versus longitude for picks located south of the SYGR and the Easter Microplate. The regression (dashed blue line) is fitted to the picks on the Nazca Plate between 0 and 15.0945 Ma (C5Bn.2n). The corresponding regression on the Pacific side covers 0 to 25.951 Ma (C8n.1n). The assigned numbers are the half-spreading rates in mm/year resulting in an average of 6.4% asymmetric accretion to the Nazca Plate and an EPR migration rate of 93.6 mm/yr. **C**) Magnetic anomaly ages versus longitude for picks located north of the SYGR and the Easter Microplate. Regressions on both the Pacific and Nazca sides of the EPR (solid blue lines) are fitted to the interval 0 to 17.466 Ma (C5Dn). The half-spreading rates in mm/year are provided, indicting an average of 10.9% asymmetric accretion to the Nazca Plate and an EPR migration rate of 89.9 mm/yr. Black vertical dashed lines illustrate the relationship between the youngest anomaly picks for the Mendoza Rise and the location of the eastern edge of the Easter plume as defined by the SYGR age progression (see Figure 9 for additional details).

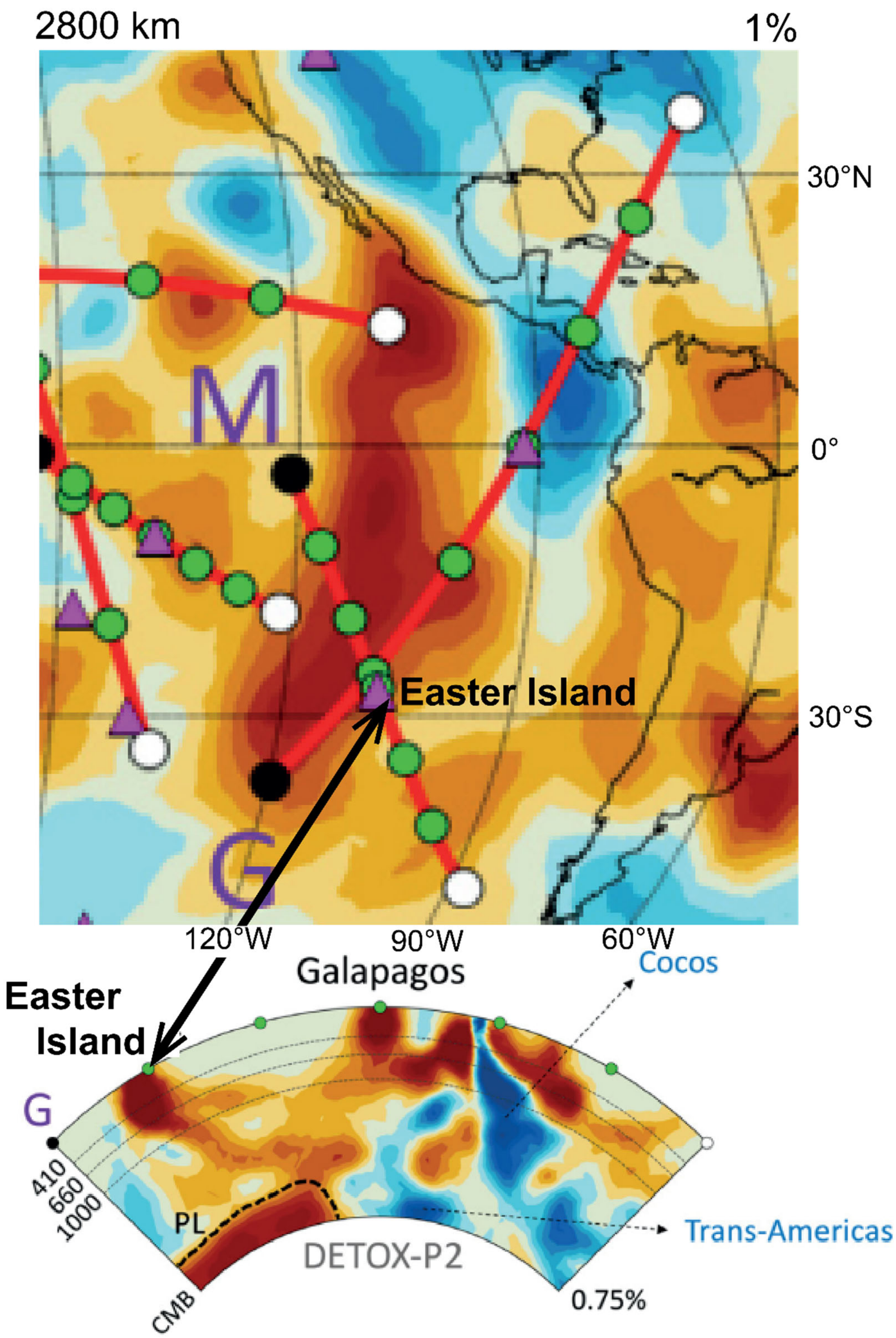

Figure S2. DETOX-P2 at 2800 km centred on the Pacific LLVP and a vertical section (profile G) through the study area. Dashed lines mark the edges of the Pacific LLVP (PL) for visual reference. Triangles denote the Galapagos and Easter plumes. Colours indicate P-velocity anomalies with respect to IASP91. Figure adapted from Hosseini et al. (2020).
