# Supplementary material for: Synchronous motion of the Easter mantle plume and the East Pacific Rise
Source: Nat Commun. 2024 Nov 17;15:9953. doi: 10.1038/s41467-024-54115-2 (PMC11570642; doi:10.1038/s41467-024-54115-2)
Supplement: Supplementary file 3 — Description of Additional Supplementary Files [file 41467_2024_54115_MOESM3_ESM.pdf]

<u>Description of Additional Supplementary Files</u>

Supplementary Data 1: Summary ArArCalc data files

Supplementary Data 2: Full ArArCalc data files.

Supplementary Data 3: Table S1. Summary of $^{40}Ar/^{39}Ar$ isotopic dating results

Supplementary Data 4: Table S2. Results of $^{40}Ar/^{39}Ar$ incremental heating experiments

Supplementary Data 5: Table S2. Trace element and Sr-Nd-Pb isotope geochemistry
